# Anti-HIV-1 Activity of a New Scorpion Venom Peptide Derivative Kn2-7

**DOI:** 10.1371/journal.pone.0034947

**Published:** 2012-04-19

**Authors:** Yaoqing Chen, Luyang Cao, Maohua Zhong, Yan Zhang, Chen Han, Qiaoli Li, Jingyi Yang, Dihan Zhou, Wei Shi, Benxia He, Fang Liu, Jie Yu, Ying Sun, Yuan Cao, Yaoming Li, Wenxin Li, Deying Guo, Zhijian Cao, Huimin Yan

**Affiliations:** 1 State Key Laboratory of Virology, College of Life Sciences, Wuhan University, Wuhan, People's Republic of China; 2 State Key Laboratory of Virology, Wuhan Institute of Virology, Chinese Academy of Sciences, Wuhan, People's Republic of China; University of Central Florida College of Medicine, United States of America

## Abstract

For over 30 years, HIV/AIDS has wreaked havoc in the world. In the absence of an effective vaccine for HIV, development of new anti-HIV agents is urgently needed. We previously identified the antiviral activities of the scorpion-venom-peptide-derived mucroporin-M1 for three RNA viruses (measles viruses, SARS-CoV, and H5N1). In this investigation, a panel of scorpion venom peptides and their derivatives were designed and chosen for assessment of their anti-HIV activities. A new scorpion venom peptide derivative Kn2-7 was identified as the most potent anti-HIV-1 peptide by screening assays with an EC_50_ value of 2.76 µg/ml (1.65 µM) and showed low cytotoxicity to host cells with a selective index (SI) of 13.93. Kn2-7 could inhibit all members of a standard reference panel of HIV-1 subtype B pseudotyped virus (PV) with CCR5-tropic and CXCR4-tropic NL4-3 PV strain. Furthermore, it also inhibited a CXCR4-tropic replication-competent strain of HIV-1 subtype B virus. Binding assay of Kn2-7 to HIV-1 PV by Octet Red system suggested the anti-HIV-1 activity was correlated with a direct interaction between Kn2-7 and HIV-1 envelope. These results demonstrated that peptide Kn2-7 could inhibit HIV-1 by direct interaction with viral particle and may become a promising candidate compound for further development of microbicide against HIV-1.

## Introduction

Nearly 34 million people were living with human immunodeficiency virus (HIV) at the end of 2010 in the globe [1] and half of them were women. Unfortunately, there are still no effective vaccine or other countermeasure to eliminate HIV transmission [2]. The Merck STEP [3] and the Thai RV144 HIV vaccine [4] trials confirmed that we still have a long way to go before developing a prophylactic HIV vaccine. Meanwhile, HIV virus spreads fast and the HIV/AIDS pandemic still stands as a serious public health problem worldwide [1]. Current situation clearly indicates the necessity of developing new anti-HIV agents which can be used for prevention of HIV/AIDS dissemination.

HIV-1 initially infects T cells through CD4 receptor [5] and either of the two chemokine co-receptors CXCR4 (X4) or CCR5 (R5) (or both) [6], [7], [8]. It has been suggested that R5 is the major co-receptor involved in sexual transmission of HIV-1 [6]. Some microbicides tested can indeed inhibit infection by X4-tropic HIV-1 but insufficiently inhibit R5-tropic HIV-1 to the same extent [9], [10]. SPL7013 is a dendrimer which had HIV-1 virucidal activity against X4 and R5X4 HIV-1 strains but not R5 virus strains [10]. Effectiveness, safety and broad spectrum are very important to an anti-HIV microbicide. The chemokine analogue PSC-RANTES had strong inhibition activity at R5-tropic HIV strains but might induce local inflammation [11]. The sulfonated polymer PRO2000 is safe but cannot provide efficacious protection against sexual HIV transmission [12]. Polyanioun had been suggested as potential microbicides [13], [14], [15]. Unfortunately, a recent phase III trial of cellulose sulfate was terminated because of its increasing rate of HIV infection than women using a placebo [16]. Therefore, more sources of antiviral reagent to prevent HIV-1 transmission are needed for efficient protection of our body from HIV infection.

Natural antimicrobial peptides (AMPs) are widely expressed and rapidly induced on epithelial surfaces to repel invasion from diverse infectious agents including bacteria, viruses, fungi and parasites [17], [18]. So far, more than 1700 AMPs of different origins have been identified or predicted [19]. Most AMPs maintain certain common features such as being small (10–50 amino acids), containing positive charge of 2 to 9 and an amphipathic structure [20], [21], [22], [23]. Scorpion venom is a cocktail of peptides and proteins with diverse bioactivities, which represent a tremendous potential for use in drug design and development [24], [25], [26]. AMPs from scorpion venom such as hadrurin [27], scorpine [28], opistoporins, parabutoporin [29], ISCTs [30] and mucroporin [31] are paid more and more attention due to their biological activity [31], [32]. Some of these molecules have activities against viral pathogens such as junin virus, herpes simplex virus, adenovirus, rotavirus, vaccinia virus, HCV and measles virus [33], [34], [35], [36], [37].

We had reported previously that mucroporin cloned from the venom of the scorpion *Lychas mucronatus* and its optimized derivative mucroporin-M1 showed antimicrobial activity on bacteria and measles virus [31], [38]. Another scorpion peptide BmKn2 which was cloned from the venom of *Mesobuthus martensii Karsch* had also showed a strong antimicrobial activity against bacteria [39]. As a further development of BmKn2 peptide, a new peptide named Kn2-7 was designed by substituting Glycine Alanine and Serine with Lysine or Arginine (G3K, A4R, and S10R) to enhance the net positive charge and α-helix structure. In this study, five scorpion venom peptides and their derivatives were screened for their anti-HIV-1 activities, and the results showed that three of them (mocroporin-M1, BmKn2 and Kn2-7) exhibited potent anti-HIV-1 activity, in which Kn2-7 showed the highest level of anti-HIV-1 activity. So, we speculated that Kn2-7 might be a potential lead peptide and was tested for antiviral effects by using a standard reference panel of subtype B HIV-1 pseudotyped virus (PV) and a replication-competent strain of HIV-1 virus. The binding assay of Kn2-7 to HIV-1 PV was performed for exploring the related antiviral mechanism by Octet Red system.

## Results

### Molecular design, synthesis, purification and characterization of the peptides

Kn2-7 (FIKRIARLLRKIF) was designed based on the peptide sequence of BmKn2 (FIGAIARLLSKIF) to enhance the net positive charge of the hydrophilic side. Kn2-7 peptide contains five positive charges while BmKn2 carries only two positive charges (Table 1). Glycine, Alanine and Serine residues of BmKn2 were replaced with Lysine or Arginine (G3K, A4R, and S10R). The secondary structures were predicted by PHD method [40] on the website (http://npsa-pbil.ibcp.fr/cgi-bin/npsa_automat.pl?page=/NPSA/npsa_seccons.html), suggesting that Kn2-7 has higher α-helix percentage than BmKn2, which are 84.62% and 76.92%, respectively. Information of mucroporin, mucroporin-M1 and mucroporin-S1 were describe by our previous study [38].

**Table 1:** The charge, first and predict secondary structure of scorpion venom peptides.

Peptide	Amino Acid Sequence	Charge	Cationic(%)	Structure Prediction
BmKn2	FIGAIARLLSKIF	2	15.38	chhhhhhhhhhcc
Kn2-7	FIKRIARLLRKIF	5	38.46	chhhhhhhhhhhc
mucroporin	LFGLIPSLIGGLVSAFK	1	5.88	cccchhhhhhhhhhhhc
mucroporin-M1	LFRLIKSLIKRLVSAFK	5	29.41	chhhhhhhhhhhhhhhc
mucropotin-S1	SLIGGLVSAFK	1	9.09	ccccccceccc

h: alpha helix, c: random coil, e: extended strand.

The five peptides were successfully synthesized on an Abimed AMS 422 synthesizer by Fmoc solid-phase peptide synthesis. The purities of all five peptides showed reliable quality of more than 95%. The molecule weights measured by mass spectrometry (MS) were completely matched with the calculated molecule weights of these peptides.

### Kn2-7 was identified as the most effective scorpion venom peptide against HIV-1 PV by screening assay

**Figure 1:**
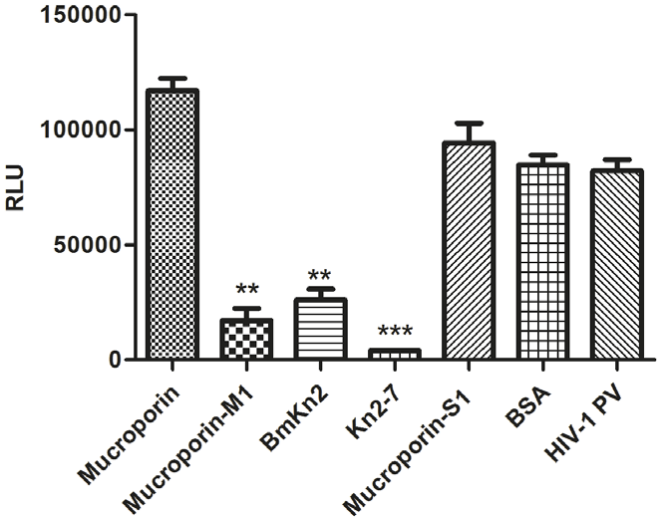
Kn2-7 was the most effective peptide against HIV-1 PV by screening assay. The antiviral effect of the peptides against HIV-1 PV was performed by TZM-bl detection systems as described in Materials and Methods. Briefly, 10 µg/ml of peptides were incubated with 200 TCID_50_ of HIV-1 PV for 1 h at 37°C and then added to TZM-bl cells. The luciferase activity was measured in a 96-well black solid plate by Turner Biosystems Modulus II microplate reader 48 h later. Significant reduction in the RLU value of groups treated by different peptides compared to virus group in which cells were exposed to BSA are denoted by a * (p<0.05), ** (p<0.01), and *** (p<0.001). Data are expressed as mean ± SD of three replicate samples.

To assess anti-HIV activities of scorpion venom peptides, pseudotyped virus-based assays with TZM-bl system were adopted for the screening of anti-HIV-1 effect of different peptides. The results showed that mucroporin-M1, BmKn2 and Kn2-7 could significantly decrease infectivity of an HIV-1 clade B b12-resistant pseudotyped virus CAAN5342 compare to the mucroporin, mucroporin-S1, BSA and no-peptide virus mock control, in which peptide Kn2-7 showed the highest level of anti-HIV-1 activity and almost completely inhibited viral infection (Fig. 1). This gave us a hint that peptide Kn2-7 is the most potential for developing anti-HIV agent. Therefore, Kn2-7 peptide was chosen for further study to clarify its anti-HIV effect. Mucroporin-S1 was chosen as a negative control for the further study because it shows no anti-bacterial and antiviral activity in our previous research [31] but has similar number of amino acid with Kn2-7 peptide.

### Kn2-7 acted sensitively and rapidly against HIV-1 PV

**Figure 2:**
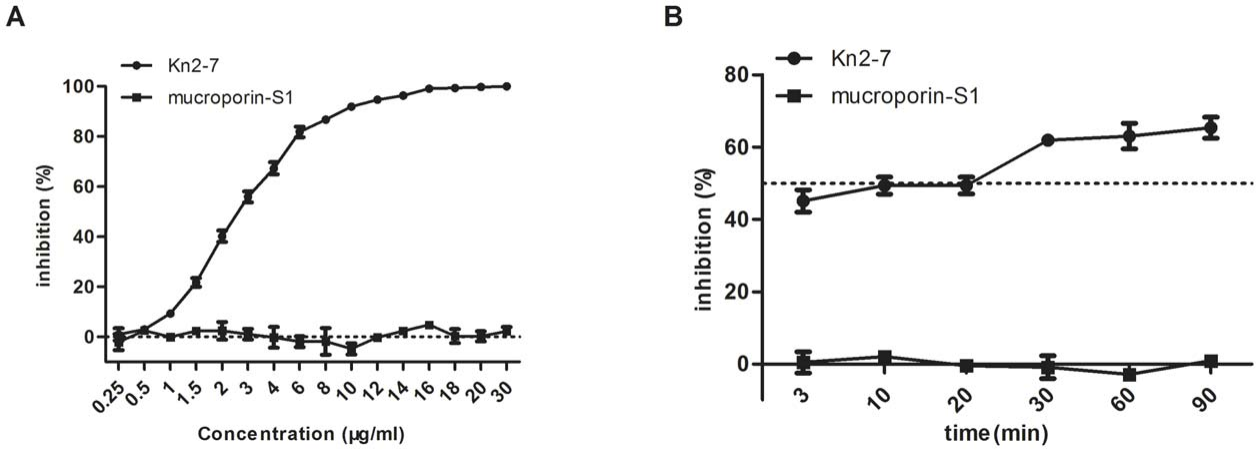
Dose- and time-dependent effects of Kn2-7 peptide against HIV-1 PV. (A) For dose-dependent effect experiments, serial dilution from 0.25 µg/ml to 30 µg/ml of Kn2-7 and mucroporin-S1 were incubated with 200 TCID_50_ HIV-1 PV for 1 h at 37°C and then added to TZM-bl cells, respectively. (B) For time-dependent effect experiments, 3 µg/ml of Kn2-7 and mucroporin-S1 were incubated with 200 TCID_50_ of HIV-1 PV for 3 min, 10 min, 30 min, 60 min and 90 min, respectively. The viruses treated without any peptide were used as the control. Data are presented as mean ± SD of three replicate samples for one representative experiment.

To confirm whether anti-HIV-1 activity of Kn2-7 was dependent on concentration, several concentrations of Kn2-7 anti-HIV-1 activity were measured by pseudotyped virus-based assays with TZM-bl detection system. As shown in Fig. 2A, the antiviral-activity of Kn2-7 against HIV-1 PV was in a dose-dependent manner and an inhibition of 40.1% was achieved at a concentration of 2 µg/ml and its EC_50_ value was calculated as 2.76 µg/ml. Up to 98.7% inhibition could be achieved when concentration rose to 16 µg/ml.

To determine the effective time needed for Kn2-7 to act on HIV-1 PV, 3 µg/ml peptide was incubated with 200 TCID_50_ of HIV-1 PV for 3 min, 10 min, 30 min, 60 min and 90 min, respectively. As shown in Fig. 2B, from 3 min to 60 min, the longer time treatment, the higher inhibition was achieved but no significant difference was observed between 60 min treatment group and 90 min treatment group. The inhibition could be up to 42.3% when Kn2-7 was incubated with HIV-1 PV for only 3 min. Therefore, the time needed for Kn2-7 to act on HIV-1 PV was very short and the activity peaked at the time point of 60 min.

According to this result, the concentration of 3 µg/ml (nearby the EC_50_ value) and 60 min treatment were chosen for further assay of Kn2-7 peptide on anti-HIV-1 PV activity in the following experiments.

### The inhibitory effect of Kn2-7 was selective to HIV-1 PV

To distinguish selective antiviral activity from nonselective cytotoxicity, we tested the cytotoxicity of the peptides on TZM-bl cells. TZM-bl cells were treated with several concentrations of Kn2-7 peptide for 24 hours and the viability of TZM-bl cells was measured using the MTS assay [41]. The absorbance was measured at 490 nm using an ELISA plate reader and the CC_50_ value were calculated by SPSS. As shown in Table 2, CC_50_ of Kn2-7 on TZM-bl cells was 38.46 µg/ml, which is 13.93 times over the EC_50_ value. Therefore, the antiviral activity of the peptide Kn2-7 against HIV-1 does not result from the cytotoxicity of the peptide on TZM-bl cells.

**Table 2:** Anti-HIV-1 PV activities and cytotoxicity of Kn2-7 peptide on TZM-bl cells.

	EC_50_ a(µg/ml)	CC_50_ a(µg/ml)	SI
Kn2-7	2.76	38.46	13.93
mucroporin-S1	I	>100	

EC_50_: peptide concentration required to reduce virus infection by 50%; CC_50_: peptide concentration required to reduce cell viability by 50%; SI (selectivity index) = CC_50_/EC_50_; I: inactive.

a All the data represent mean values for at least three independent experiments.

### Kn2-7 directly targeted HIV-1 PV

**Figure 3:**
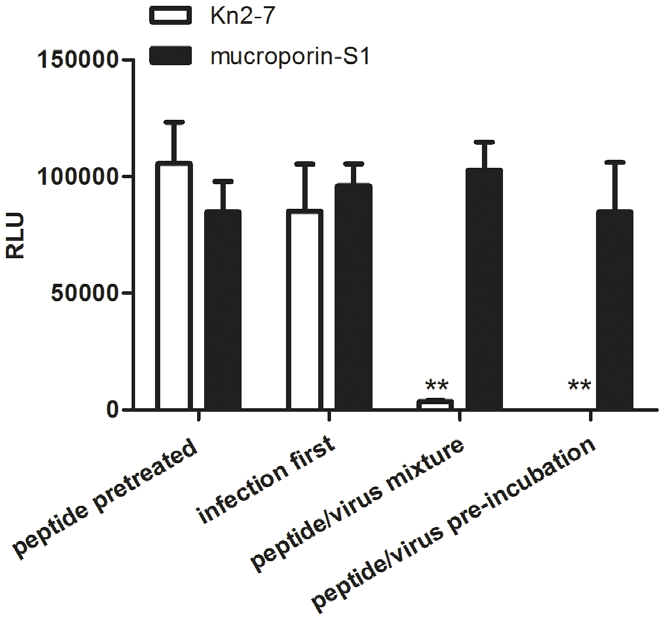
Directly inactivated mechanism of Kn2-7 peptide to HIV-1 PV. Four action modes of treatment between peptides with HIV-1 PV were carried out. (1) Peptide pretreated group, TZM-bl cells were pretreated with 10 µg/ml peptide at 37°C for 1 h and then treated with HIV-1 PV. (2) Infection first group, cells were infected with HIV-1 PV first by inoculating at 37°C for 1 h and then treated with 10 µg/ml peptide. (3) Peptide/virus mixture group, cells were treated with mixture of HIV-1 PV and 10 µg/ml peptides immediately after mix but without inoculation. (4) Peptide/virus pre-incubation group, cells were infected with mixture of HIV-1 PV and 10 µg/ml peptides pre-incubating at 37°C for 1 h. The inhibitory effects of different groups were determined in TZM-bl detection system. Significant reduction in the RLU value of Kn2-7 treatment group compared with that of mucoporin-S1 treatment group are denoted by a * (p<0.05), ** (p<0.01), and *** (p<0.001).

In order to gain some insight into the mechanism of anti-HIV-1 activity of Kn2-7, the interaction time and order among Kn2-7, HIV-1 PV and TZM-b1 cells were varied by four different ways described in Materials and Methods. Mucroporin-S1 were prepared in the same manner and set as mock group. As shown in Fig. 3, Kn2-7 incubating with HIV-1 PV for 1 h before inoculating to TZM-b1 cells (peptide/virus pre-incubation group) showed prominent inhibitory effect, agreed with the results above. Although in a low degree, Kn2-7 and HIV-1 PV adding to cells simultaneously but without pre-incubation (peptide/virus mixture group) also showed a significant inhibition. However, neither the peptide pretreated group, in which TZM-bl cells were pre-treated with Kn2-7 for 1 h, nor the infection first group, in which TZM-bl cells were infected with HIV-1 PV first and then treated with Kn2-7 showed any antiviral effect (Fig. 3).

**Figure 4:**
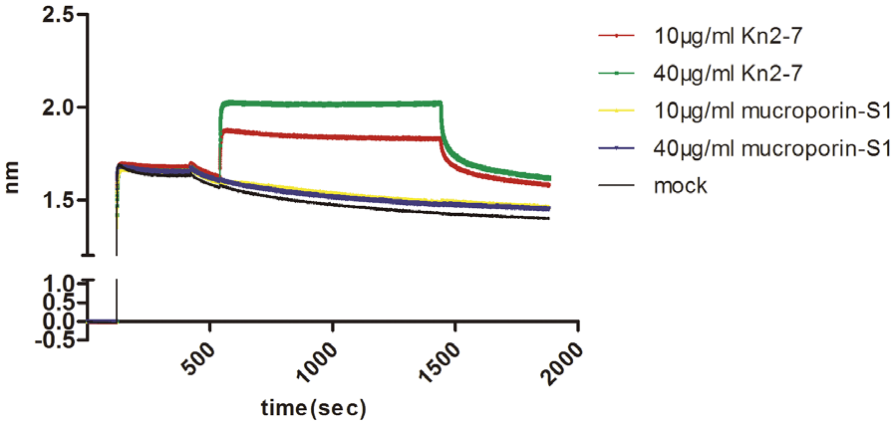
Direct binding of peptide Kn2-7 to HIV-1 PV. HIV-1 PV was bound and analyzed, in parallel, by five Octet APS sensors. APS sensors were pre-wet for 120 s, and then coupled with 200 µl HIV-1 PV for 300 s. After clearance of unabsorbed PV, APS sensors were associated with 10 µg/ml and 40 µg/ml of Kn2-7 or mucroporin-S1 for 900 s (Measured in nanometers).

Based on the results of four treatment modes of Kn2-7, we assumed that the anti-HIV activity of Kn2-7 attributed to its interaction with HIV-1 viral particle directly. To confirm the hypothesis, a binding assay based on Biolayer Interferometry was performed to study the interaction between Kn2-7 and HIV-1 PV. HIV-1 PV was loaded to APS biosensor and the sensor tip was transferred to Kn2-7 or mucroporin-S1 with concentration of 10 µg/ml or 40 µg/ml. Expectedly, we observed rapid direct binding between HIV-1 PV and Kn2-7 and the binding capacity of 40 µg/ml Kn2-7 was stronger than that of 10 µg/ml. Mucroporin-S1 showed no significant binding with HIV-1 PV (Fig. 4). Therefore, Kn2-7 peptide executes anti-HIV-1 activity by direct targeting HIV-1 particle.

### Kn2-7 had broad-spectrum of antiviral activity against HIV-1 PV

**Figure 5:**
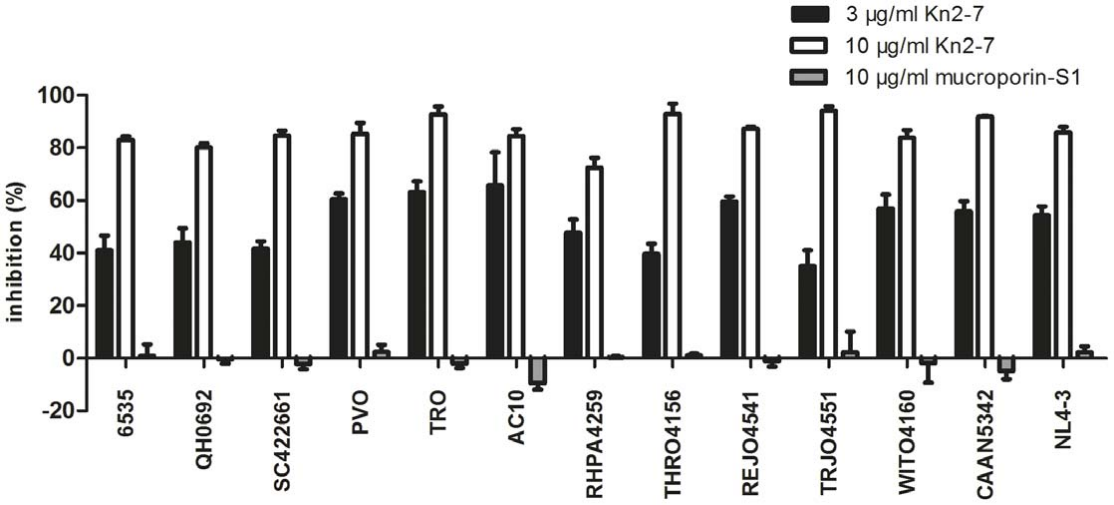
Broad-spectrum antiviral activity of Kn2-7 against 13 strains of HIV-1 subtype B PV. The inhibitory effect of the peptide was determined in TZM-bl luciferase detection system. Briefly, 3 µg/ml and 10 µg/ml of Kn2-7 was incubated with different strains of HIV-1 PV (200 TCID_50_) for 1 h at 37°C and then added to TZM-bl cells. The group treated with mucroporin-S1 and without any peptide in the same manner served as the negative and background control, respectively.

To define whether the anti-viral effect of Kn2-7 peptide against HIV-1 PV is broad-spectrum or not, the Kn2-7 (3 µg/ml and 10 µg/ml) against a standard reference panel of HIV-1 subtype B isolates were measured using TZM-bl detection system and mucroporin-S1 was used as the negative control. Inhibitions were calculated compared to the HIV-1 PV control group. 3 µg/ml and 10 µg/ml of Kn2-7 peptide showed comparable antiviral potencies against 13 strains of HIV-1 subtype B PV including 12 R5-tropic (6535, QH0692, SC422661, PVO, TRO, AC10, PHPA4259, THRO4156, REJO4551, TRJO4551, WITO4160, CAAN5342) and 1 X4-tropic (NL4-3). The negative control of mucroporin-S1 show no inhibitory effect at corresponding concentrations (3 µg/ml of mucroporin-S1 data not shown) (Fig. 5). Consequently, Kn2-7 peptide had a broad-spectrum antiviral activity against HIV-1 PV regardless of tropism.

### Kn2-7 had anti-viral effect against replication-competent HIV-1 virus

**Figure 6:**
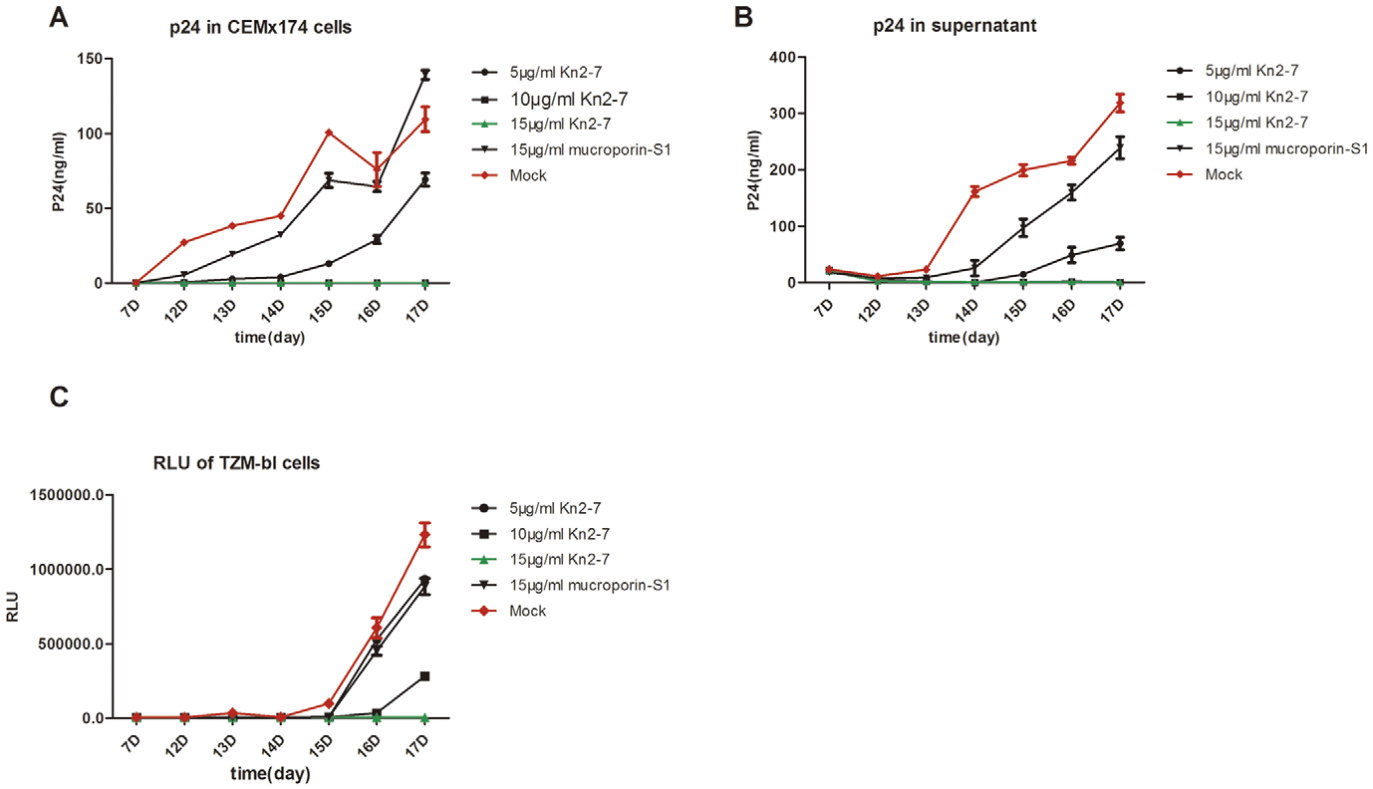
Antiviral activity of Kn2-7 against a replication-competent HIV-1 virus. Three dilutions of Kn2-7 or mucroporin-S1 were incubated with HIV-1 virus for 1 h at 37°C, and then were added to CEM×174 cells in 12-well plates in duplicates for 17 days. Samples were collected at day 7, 12, 13, 14, 15, 16, and 17 after infection and p24 protein concentration of the cells and supernatants were detected by ELISA. The infectivity was measured by TZM-bl detection luciferase assay. (A) CEM×174 cells samples were measured for p24 protein by ELSIA. (B) CEM×174 supernatant samples were measured for p24 protein by ELSIA. (C) Samples were detected for infectivity by TZM-bl detection system.

To confirm the potent inhibition of Kn2-7 to HIV-1, a replication-competent HIV-1 virus was also adopted for the anti-HIV activity assay. Serial dilutions of Kn2-7 or mucroporin-S1 were incubated with 20 µl X4-tropic proviral DNA (pKS242) derived HIV-1 virus (15 ng p24) for 1 h at 37°C and then infected CEM×174 cells for 17 days. Supernatant and cell samples were collected at day 7, 12, 13, 14, 15, 16 and 17. P24 antigen expression was analyzed by ELISA and infectivity was further detected by TZM-bl detection system. 10 µg/ml and 15 µg/ml of Kn2-7 suppressed p24 expression completely from 7^th^ day to 17^th^ day in infected cell (Fig. 6A) as well as in supernatant (Fig. 6B). 5 µg/ml of Kn2-7 suppressed p24 expression in cell sample in early stage (7^th^ day to 14^th^ day), but not in the later time points, with the value of p24 rose at 15^th^ day, till to 17^th^ day (Fig. 6A). The p24 antigen level in supernatants showed the same trend as that of cell samples by 5 µg/ml of Kn2-7, but with a delayed reaction (Fig. 6B). The negative control peptide, mucroporin-S1, did not show any inhibitory effect. To further determine antiviral activity of Kn2-7, the above samples was added to TZM-bl cells for detection of their infectivity. Luciferase activities of infected TZM-bl cells were measured after 48 h incubation. Similar trends of inhibitory effect were observed (Fig. 6C). However, 15 µg/ml of Kn2-7 inhibited virus replication completely but 10 µg/ml of Kn2-7 did not (Fig. 6C) while both 10 and 15 µg/ml of Kn2-7 showed completed suppression by p24 assay (Fig. 6A, 6B). The results further demonstrated that the Kn2-7 can inhibit HIV infectivity and its consequent replication. To rule out potential inhibitions attributable to cytotoxicity to host cells, the cytotoxicity of the peptides on CEM×174 cells were measured by using the MTS assay from 5 µg/ml to 100 µg/ml and the CC_50_ value of Kn2-7 on CEM×174 cells was 43.18 µg/ml, which shown that the antiviral activity of Kn2-7 against HIV-1 does not result from the cytotoxicity of the peptide on CEM×174 cells.

In brief, the scorpion venom peptide derivative Kn2-7 not only significantly inhibits HIV-1 PV, but also has antiviral effect against replication-competent HIV-1 virus with broad-spectrum by direct interaction with viral particle and may become a promising candidate compound for further development of anti-HIV-1 agent.

## Discussion

For three decades, HIV/AIDS has wreaked havoc on the population of the world. Although the number of new infections has been falling, levels of new infections overall are still high. The vast majority of people newly infected with HIV are infected during unprotected sexual intercourse [42]. The quest for effective microbicide and drug to prevent initial HIV-1 infection and treat AIDS patients is essential for countering the spread of the diseases worldwide when there is no effective prophylactic HIV vaccine available at present. Tremendous efforts have been directed towards development of effective anti-HIV-1 microbicides, which are defined as products that can be applied topically for the prevention of HIV-1 and other sexually transmitted infections by creating chemical, biological and-/or physical barriers. An effective microbicide against HIV should be efficient with anti-HIV-1 activity, safe with low cytotoxicity to host cells and broad-spectrum against both R5-tropic and X4-tropic HIV-1 [6],[43],[44]. For satisfying all the criteria above, more resources of possible anti-HIV-1 reagents should be explored.

In previous studies, we isolated several kinds of cationic antimicrobial peptides from the cDNA libraries of the venomous gland of scorpions and identified their anti-bacterial activity against pathogens [24], [31]. Recently we reported that mucroporin-M1, one kind of modified scorpion venom peptide, can inhibit some RNA viruses such as measles virus, SARS-CoV and influenza H5N1 viruses [38]. In this study, we screened the anti-HIV-1 efficacy of five scorpion venom peptides and their derivatives. The results showed that Kn2-7, BmKn2 and mucorporin-M1 exhibited varying degrees of antiviral effects and among them Kn2-7 peptide was the most effective with an EC_50_ value of 2.76 µg/ml (Fig. 1). According to the most potent anti-HIV-1 activity and low toxicity on TZM-bl cells and the mouse skin model (unpublished data), Kn2-7 was chosen for further investigation to clarify its anti-HIV-1 effect. Kn2-7 was modified from BmKn2 by enhancing the net positive charge of the hydrophilic side and the amphiphilic helix through amino acid replacing (G3K, A4R, and S10R). We found Kn2-7 had higher anti-HIV-1 activity than BmKn2, but with similar CC_50_ to cells and even decreased toxicity response by the mouse skin model test (unpublished data). So, the modification improved the specific anti-viral activity in a certain extend. However, the mechanism needs to study further.

Since CCR5 is the major co-receptor involved in sexual transmission which becomes the leading infection route of HIV-1, antiviral activity against R5-tropic HIV-1 is more important [6], [43]. Therefore, CAAN5342, a R5-tropic HIV-1 PV, the most tolerated HIV-1 PV to monoclonal antibody B12 and 2G12 in the standard reference panel of HIV-1 subtype B pseudotyped virus was chosen to test the antiviral activity of Kn2-7 [45]. Kn2-7 showed potent antiviral activity against CAAN5342 in a dose- and time-dependent manner (Fig. 2). Further studies demonstrated that Kn2-7 was effective against HIV-1 subtype B PV regardless of their tropism with a selective index (SI) of 13.93. It was also highly effective to suppress replication-competent HIV-1 strain (Fig. 6). The results demonstrated that Kn2-7 was broad-spectrum against HIV-1 which was in accordance with our previous findings that scorpion venom peptides are broad-spectrum biocides [24], [31], [38].

On the other hand, Kn2-7 was proven to be active to both cell-free and cell-associated replication-competent HIV-1 exhibited by its suppressing capability on infected TZM-bl cells, in which 15 µg/ml of Kn2-7 almost completely inhibited viral replication (Fig. 6C). As shown by Cole et al., HIV-1 evolves little resistance during continued passaging in the presence of defensins, another kind of cationic antimicrobial peptide [46]. Kn2-7 may also possess this particular property which can reduce the likelihood of the emergence of resistance and the subsequent spread of infection.

As reported earlier, scorpion venom peptides display anti-pathogen activity by breaking bacteria directly [31], [38]. We assumed that Kn2-7 may implement the inhibition of HIV-1 with the similar mechanism. The experiment of four different treatment groups of Kn2-7 to HIV-1 PV, in which the viral infectivity was inhibited only when Kn2-7 had chance to interact directly to HIV-1 PV, gave us a hint that Kn2-7 may exert its antiviral activity by damaging HIV virus particle itself. To clarify the presumption, we tried the binding assay for possible interaction between Kn2-7 and HIV-1 PV with Octet Red system by using APS sensors. The result confirmed that Kn2-7 could directly bind to HIV-1. However, whether viral particle could be broken by this binding is not clear. In experiments for effective action time of Kn2-7 to HIV-1 PV, it was observed that 3 min interaction allowed Kn2-7 showing almost maximum inhibition to HIV-1 PV (Fig. 2B). This told us that the anti-HIV-1 activity of Kn2-7 was rapid and direct which is similar to the action manner of mucroporin to bacteria [31]. Barrel-stave model might explain what happened between Kn2-7 and HIV-1 virus to some extent [47], [48], [49]. We may deduce the action principles as following: once Kn2-7 encounters with HIV-1, Kn2-7 binds to the envelope of HIV-1 directly and immediately. The attached Kn2-7 aggregates and inserts into viral envelope so that the hydrophobic peptide regions align with the lipid core region and the hydrophilic peptide regions form the interior region of the pore, with the help of positive charge of peptide somehow. However, more investigations need to be done for further clarification.

The epidemic of HIV-1 combined with the lack of an effective vaccine urge us to find more new resources of antiviral agents to help control HIV-1 transmission. In this study, a rationally designed peptide, Kn2-7, shows potent antiviral activities against 13 strains of HIV-1 subtype B strains by direct interaction with virus regardless of their tropism with low cytotoxicity. The data presented here suggest that Kn2-7 is a potential microbicide candidate against HIV-1 virus and even become a powerful anti-HIV-1 drug component when it is conjugated with HIV-1 specific antibodies. All in all, scorpion venom peptides can be at least served as molecular templates for designing antiviral peptides for HIV-1. Further pharmacokinetic, pharmacodynamic, and toxicological studies in animal models are worth being conducted to define safety parameters and application potential for the practical use of Kn2-7 as preventive agents for HIV-1 transmission.

## Materials and Methods

### Molecular design, synthesis, purification and characterization of scorpion venom peptides

BmKn2 is a 13 amino acid antimicrobial peptide which cloned from the venom of *Mesobuthus martensii Karsch* [39]. Kn2-7 is an optimizing design of BmKn2 by substituting Glycine Alanine and Serine with Lysine or Arginine (G3K, A4R, and S10R). Mucroporin is a 17 amino acid peptide from the venom of the scorpion *Lychas mucronatus*. Mucroporin-M1 is a derivative of mucroporin by substituting Glycine and Proline residues with Lysine or Arginine (G3R, P6K, G10K, and G11R) [31]. Mucroporin-S1 is an 11-amino peptide by removing 6 N-terminal amino acids from mucroporin as a control in this study.

Kn2-7 (FIKRIARLLRKIF), BmKn2 (FIGAIARLLSKIF), mucroporin (LFGLIPSLIGGLVSAFK), mucroporin-M1 (LFRLIKSLIKRLVSAFK), and mucroporin-S1 (SLIGGLVSAFK), were synthesized on an Abimed AMS 422 synthesizer by Fmoc solid–phase peptide synthesis. The purification and characterization were carried out as described before [38]. Briefly, all peptides were purified on a C18 column (Elite HPLC, China) using a linear gradient and peaks were detected by the absorbance at 215 nm and collected manually. Thereafter, peptides were lyophilized and the purity was tested by high-performance liquid chromatography (HPLC) and mass spectrometry.

### Cells

TZM-bl cells (NIH AIDS Research and Reference Reagent Program, Division of AIDS, NIAID, National Institutes of Health) are a genetically engineered HeLa cell clone that express CD4, CCR5 and CXCR4, and was further engineered with an HIV-1-based vector to contain Tat-responsive reporter genes for firefly luciferase (Luc) as contributed by John Kappes and Xiaoyun Wu [50],[51]. 293 T, Vero and CEM×174 cell lines were obtained from American Type Culture Collection (Rockville, MD, U.S.). TZM-bl, 293 T and Vero cells were cultured in Dulbecco's modified Eagle's medium (Gibco DMEM) containing 10% fetal bovine serum (FBS, 56°C heat-inactivated, Gibco), 1% L-glutamine and 1% penicillin/streptomycin (Gibco). The cells were incubated at 37°C in a humidified atmosphere containing 5% CO_2_. The CEM×174 cells were cultured in RPMI 1640 medium (Gibco) supplemented with 10% FBS, 1% L-glutamine, and 1% penicillin/streptomycin.

### Preparation and titration of single-cycle HIV-1 PV and replication-competent HIV-1 virus from proviral DNA

All clones for pseudotyped virus-based assays were provided by NIH AIDS Research and Reference Reagent Program. Stocks of Env-pseudotyped virus were produced in 293 T cells briefly as following. 293 T cells (5×10^6^ cells/ml) were co-transfected with 6 µg of an Env-deficient HIV-1 backbone vector (pSG3ΔEnv) and 4 µg of a plasmid encoding envelope protein of one strain of HIV-1 subtype B (6535, QH0692, SC422661, PVO, TRO, AC10, PHPA4259, THRO4156, REJO4551, TRJO4551, WITO4160, CAAN5342 [45], [50], [52], NL4-3 [53]) using Lipo2000 agent (Invitrogen) in 6-well plates. PV-containing culture supernatants were harvested 48 h after transfection, centrifuged, purified by 0.45 µm filter and stored at −80°C in 200 µl aliquots. The 50% tissue culture infectious dose (TCID_50_) measurements were done in triplicates using 8 serial four-fold dilutions of each HIV-1 PV. For each dilution, every well of a 96-well culture plate was added with 100 µl diluted PV firstly and then with trypsinized TZM-bl cells (10^4^ cells in 100 µl of growth medium containing 15 µg/ml DEAE-dextran (Sigma)). Plates were incubated at 37°C in a humidified, 5% CO_2_ atmosphere for 48 h. To measure luciferase activity, culture medium was removed, monolayers were washed once with 200 µl PBS, and 100 µl cell culture lysis buffer (Promega) was added to cells and incubated for 20 min at room temperature to allow cell lysis. After that, 50 µl of cell lysate was transferred to 96-well black solid plate (Promega) and relative luminescence units (RLU) activity was measured by a Luciferase Assay System according to the manufacturer's instructions (Promega) using Tuner Biosystems Modulus II. Samples producing relative light units (RLU)>2.5×background wells (containing cells only) were scored as positive infection. An endpoint virus titer of TCID_50_ was calculated by the Spearman-Karber method [54].

Except in broad-spectrum antiviral effect assay, a panel of HIV-1 PV (R5-tropic (6535, QH0692, SC422661, PVO, TRO, AC10, PHPA4259, THRO4156, REJO4551, TRJO4551, WITO4160, CAAN5342) and X4-tropic (NL4-3) HIV-1 PV) was used, a R5-tropic HIV isolate CAAN5342 was used in all other pseudotyped virus-related experiments.

An HIV-1 subtype B, X4-tropic proviral DNA (pKS242 molecular clone) [55] was used to produce replication-competent virus by transfecting Vero cells with Lipo2000 agent, and virus was propagated in CEM×174 cells. Cultures were maintained and HIV-containing CEM×174 cells and supernatant were collected. HIV-1 replication-competent viruses were quantified by p24 concentration.

### Cell viability assay

*In vitro* cytotoxicity test of Kn2-7 and mucroporin-S1, were performed by 3-(4, 5-dimethylthiazol-2-yl)-5-(3-carboxymethoxyphenyl)-2-(4-sulfophenyl)-2H-tetrazolium) (MTS) procedure [41]. Briefly, TZM-bl or CEM×174 cells were seeded into 96-well plates at a density of 1×10^4^ cells/well in triplicates and incubated at 37°C for 24 h. Thereafter, culture medium was replaced by 100 µl serial dilutions of the peptides (from 5 µg/ml to 100 µg/ml) in DMEM culture, and the cells were incubated for another 24 h. Each dilution was tested in triplicates and all the experiments were repeated three times. Then 20 µl of CellTiter 96® AQueous One Solution Reagent (Promega) was added to each well of the 96-well assay plate containing 100 µl sample in culture medium. The plate was further incubated for 2 h at 37°C in a humidified, 5% CO_2_ atmosphere. The absorbance was measured at 490 nm using an ELISA plate reader (Multiskan MK3, Thermo Labsystems). The relative cell viability (%) related to control wells containing cell culture medium without peptide was calculated by [A] test/[A] control×100%. The cytotoxicity of each peptide is expressed as the 50% of cytotoxic concentration (CC_50_), which is the concentration that inhibits the growth of 50% of cells relative to non-treated control cells.

### Antiviral effect assay of the peptides against HIV-1 PV

To test the antiviral effect of the peptides against HIV-1, pseudotyped virus-based assays with TZM-bl system were performed as described previously [56]. Briefly, 200 TCID_50_ of HIV-1 PV were incubated with indicated concentration of test scorpion venom peptides in a total volume of 100 µl for 60 min or indicated time intervals when considered with time-course effects at 37°C in triplicates in 96-well plates. After incubation, freshly trypsinized TZM-bl cells (10^4^ cells in 100 µl of growth medium containing 15 µg/ml DEAE-dextran) were added to each well and incubated for 48 h. The luciferase activities of the TZM-bl cells were measured for antiviral activities of tested peptides, in which virus control and blank control were set up as wells of cells plus virus and cells only respectively.

In screening assay of anti-virus effect, 10 µg/ml peptides were used. For the following anti-HIV-1 PV effect assay, 3 µg/ml of the lead peptide Kn2-7 and the truncated control peptide mucroporin-S1 were used except for the dose-dependent effect experiment, in which serial dilutions from 0.25 µg/ml to 30 µg/ml were used.

In the time-dependent effect experiment, 3 µg/ml of peptides were incubated with 200 TCID_50_ of HIV-1 PV for 3 min, 10 min, 30 min, 60 min, and 90 min, respectively.

### Measurement of antiviral activity of the peptides against replication-competent HIV-1 virus

Three dilutions of peptide (5 µg/ml, 10 µg/ml, and 15 µg/ml) were incubated with HIV-1 replication-competent virus (15 ng p24) for 1 h at 37°C. Thereafter, the compounds were added to CEM×174 cells (10^5^ cells/ml) in duplicates in 12-well plate. Samples were collected at day 7, 12, 13, 14, 15, 16, and 17 after infection. Supernatant and cell samples were separated by centrifuge 1000 rpm for 5 min (Thermo). Samples were detected for p24 by ELISA and measured for infectivity by TZM-bl detection systems as described above.

### Action mode of the peptides to HIV-1 PV

In order to help determine the possible antiviral mechanism of Kn2-7 peptide, four different treatment groups were designed for comparison of the antiviral effect as following: (1) peptide pretreated group, TZM-bl cells were pretreated with 10 µg/ml peptide at 37°C for 1 h and then treated with HIV-1 PV; (2) infection first group, TZM-bl cells were infected with HIV-1 PV first by inoculating at 37°C for 1 h and then treated with 10 µg/ml peptide; (3) peptide/virus mixture group, TZM-bl cells were treated with mixture of HIV-1 PV and 10 µg/ml peptides immediately after mix but without inoculation; and (4) peptide/virus pre-incubation group, TZM-bl cells were infected with mixture of HIV-1 PV and 10 µg/ml peptides pre-incubating at 37°C for 1 h. To compare the effects of peptide to viral infection, TZM-bl cells were measured by luciferase activity assay after 48 h incubation as described above. 200 TCID_50_ of HIV-1 PV (CAAN5342) was used in different treatment groups. In parallel, mucroporin-S1 control groups were set up in the same four manners.

### p24 protein detection by ELISA

For p24 protein detection, a double antibody sandwich ELISA was used [57]. Briefly, ELISA plates (Greiner Bio-one Microplate) were coated with rabbit anti-p24 polyclone-antibody (3 µg/ml in carbonate buffer 0.1 M NaHCO_3_, 0.1 M Na_2_CO_3_, pH 9.6) at 37°C for 2.5 h. Thereafter, plates were blocked using 10% FBS in PBS at 4°C overnight. Samples were serially diluted with PBS containing 10% FBS and 1% (v/v) TritonX-100 and treated for 3 h at 37°C to release p24 from the HIV-1 PV particles. P24 standard samples were treated in the same way to create a standard curve. Then treated sample dilutions were placed in wells for 2 h at 37°C. The plates were washed 6 times with PBS containing 0.05% Tween 20 (PBS-T). Mouse anti-p24-HRP antibody (1∶1200) was added and incubated for 1 h at 37°C and then washed 6 times with PBS-T. 100 µl of 3, 3′, 5, 5′-tetramethylbenzidine (TMB, Sigma Aldrich) was added to each well and incubated for 10–20 minutes at room temperature depending on the degree of colorimetric conversion of standard samples. The reaction was stopped with 1 M H_2_SO_4_, and the plate was read at 450 nm and 570 nm. OD value = (OD value at 450 nm) - (OD value at 570 nm). The p24 standard curve was drawn and a linear regression equation was made. The linear range was from 5 pg/ml to 100 pg/ml and the p24 concentration of the sample was computed using the equation.

### Biolayer Interferomtery

Binding assays were performed in 96-well microplates at 25°C by Octet Red system (Ferbio) [58], [59]. All the measurement processes are under computer control. The run was carried out by placing the APS sensors in the wells and measuring changes in layer thickness (in nanometers, nm) with time. Serial dilutions of peptides (Kn2-7 and mucroporin-S1) were run in 200 µl volumes under the same assay. Firstly, APS sensors were rinsed in DMEM buffer for 120 s which served as the background buffer. Secondly, APS sensors were coupled with 200 µl culture containing HIV-1 PV for 300 s. Thirdly, APS sensors were moved into DMEM buffer and incubated for another 120 s to clear unabsorbed HIV-1 PV. Lastly, APS sensors were exposed to Kn2-7 or mucoporin-S1 at concentrations of 10 µg/ml or 40 µg/ml. Association was monitored for 900 s followed by dissociation in DMEM buffer alone for another 400 s. The standard curve was measured at the beginning and the end of the assay to confirm that it was reproducible and valid over the time taken to run all rows of samples. Data were processed automatically using the Octet User Software version 3.1.

### Data analysis

The virus titer was calculated within each individual experiment using the method of Reed and Muench [60]. All statistical analyses were conducted using GraphPad Prism, version 5. Statistical comparisons were conducted using the 2-tailed unpaired *t* test. P values <0.05 were considered significant. Data were presented as one representative of at least three repeated experiments and expressed as mean ± standard deviation (SD) of triplicates. The value of the TCID_50_, CC_50_ and EC_50_ were analog computation by SPSS statistical software version 13 (SPSS, Chicago, IL).
